# DNA methylation in cancer: three decades of discovery

**DOI:** 10.1186/gm553

**Published:** 2014-05-30

**Authors:** Andrew Feinberg

**Affiliations:** 1 Center for Epigenetics, Johns Hopkins School of Medicine, Baltimore, MD 21205, USA

## Abstract

Andrew Feinberg shares his views on the field of cancer epigenetics, from its beginnings to the most exciting recent findings.

## Introduction

**Figure 1:**
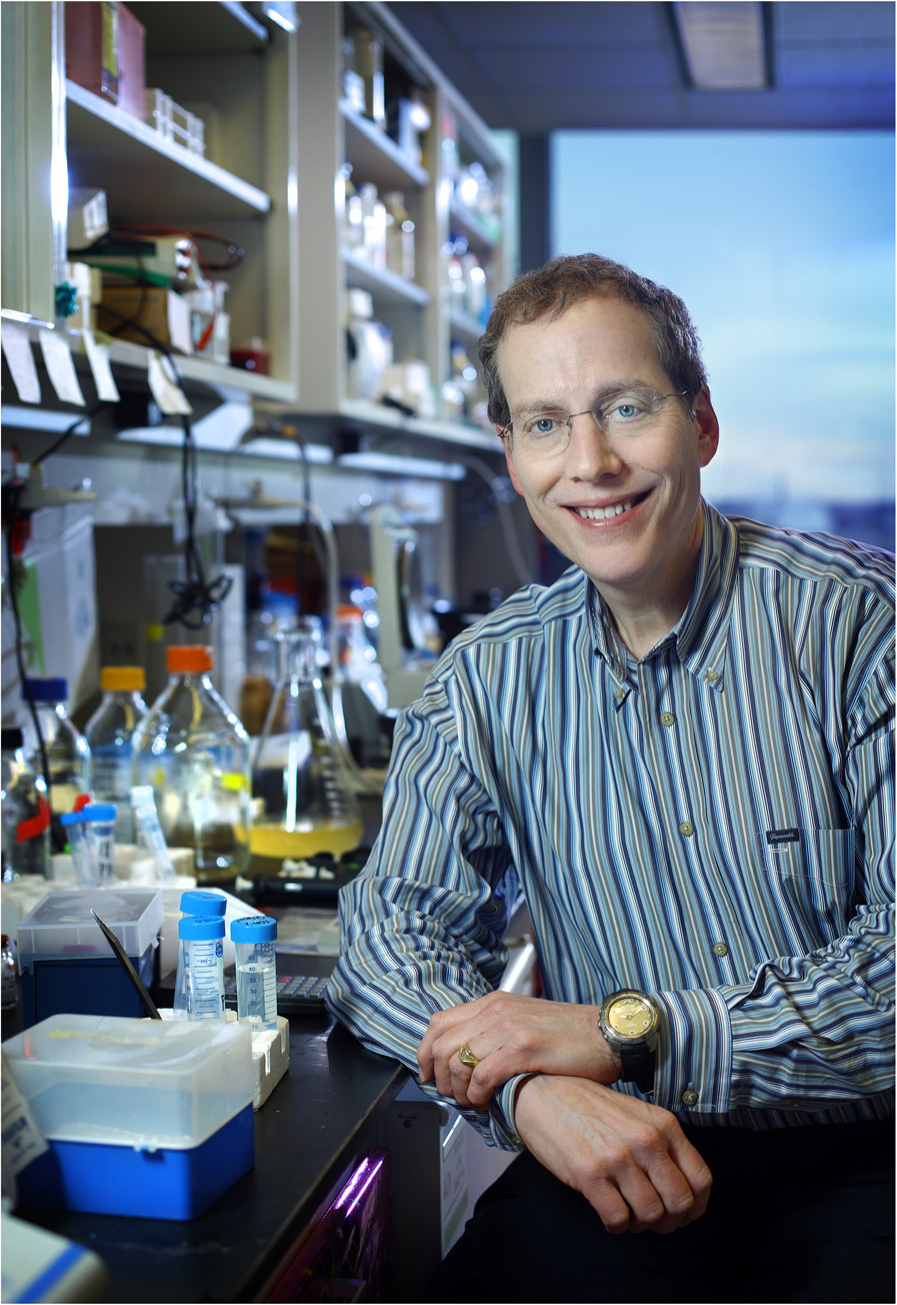
Andrew Feinberg.

Andrew Feinberg, MD, MPH (Figure 1), is Director of the Center for Epigenetics at the Johns Hopkins School of Medicine, Maryland, USA. From the beginning of the field of cancer epigenetics right through to his current work, he has been a source of pioneering ideas about how changes in DNA methylation influence cancer.

### 1) What inspired you to study the role of DNA methylation in cancer?

After medical school in the late 1970s, I did a postdoc on the slime mold *Dictyostelium discoideum*, investigating cell fate commitment and migration. I had developed a method for purifying cells that were committed to differentiation into the two mature cell types, spore and stalk, while they were still undifferentiated slug cells, but already committed to their destiny. In other words, there was a form of non-genetic memory that was stably inherited during cell division. This was an example of epigenetics, although the term was not widely used at the time.

A year later, I did an MPH (Master’s in Public Health) in quantitative areas of epidemiology, biostatistics and biomedical engineering, still looking for a research direction. During that year, I happened to hear a lecture by Don Coffey on cancer cells and tumor cell heterogeneity. After his talk, I told him that my work on *Dictyostelium* pluripotency was really the same thing that he was talking about in human cancer. There had to be some non-genetic information that was stably inherited but at the same time subject to plasticity. This led to a term paper in which I argued that the causes of cancer are both genetic and epigenetic. Don also introduced me to Bert Vogelstein, who at the time was working on DNA replication and loop topology in another slime mold, *Physarum polycephalum*. Bert kindly agreed to take me on for a second postdoc to pursue cancer genetics and epigenetics, as he was much more interested in cancer than in *Physarum*.

This was very lucky for me because I just happened to land in the lab of a wizard. We chose colon cancer as a model because of what turned out to be a mistake in someone else’s *Nature* paper. This paper presumed to show widespread gene rearrangements in colon cancer compared to matched normal mucosa of the same tissue type, but it turned out that the normal tissues had been contaminated by *Escherichia coli*. In contrast to the absence of widespread gene rearrangements, the evidence for altered DNA methylation was overwhelming. I still remember when I got the first Southern blot showing altered DNA methylation in cancer. I told Bert in the stairway to radiation oncology where we used to develop the blots because we couldn’t afford a developer. We knew we had something important - the data were just screaming off the film.

### 2) It’s more than 30 years since your *Nature* paper with Bert Vogelstein showed that some genes in human cancer cells were hypomethylated compared to their normal counterparts. What impact did that work have on the field?

Well, there was no field before that. People had not examined any epigenetic mark comparing human cancers to matched normal tissues from which they arose. We had a long conversation about how to label the figures and decided on ‘N’ (for normal) and ‘C’ (for cancer) - this became a kind of trademark through many publications. Also at the time, there was a huge unsolved problem regarding how cancers acquire properties that are normal in other cell types at other stages of development. As DNA methylation had already been linked to normal differentiation and gene expression, the study suggested a mechanism for abnormal gene expression in cancer and tumor cell heterogeneity.

### 3) Do we understand yet how altered DNA methylation contributes to cancer?

The discovery that we reported in *Nature* was about methylation changes *per se*, not just hypomethylation. The point was that changes in methylation would have effects on gene regulation. There have been many papers showing gene activation or dysregulated expression in cancer. A few years after our paper [1], Horsthemke and others [2] identified hypermethylation of tumor suppressor genes. But recent data, such as Richard Meehan’s [3], suggest that much of this epigenetic silencing is a manifestation of pre-existing chromatin changes at silenced genes in the normal tissues from which the cancer arises. Moreover, the ‘CIMP’ (CpG island methylator phenotype) seems to be directly downstream of signaling pathway mutations [4]. On the other hand, the hypomethylated domains seem to correspond to important regions of nuclear structure. We have learned a great deal more about this hypomethylation from recent whole-genome studies. The hypomethylated domains correspond to nuclear domains of heterochromatin that we identified earlier [5] and termed LOCKs (large organized chromatin lysine modifications; which others call LADs (lamin-associated domains)) or to the partially methylated domains in normal cells identified by Lister and colleagues [6]. The hypomethylation occurs very early in cancer (for example, in Epstein-Barr virus immortalized cells or in premalignant adenomas) and it leads to highly variable expression of the genes that are activated. We think, therefore, that disrupted methylation is a major factor in tumor cell heterogeneity, which is what motivated me to study this area decades ago.

Winston Timp, Rafa Irizarry and I are working on the possible relationship between the local small changes in methylation - hypomethylated shores and hypermethylated islands - and the large hypomethylated block domains. In other work, we have found that epithelial mesenchymal transition involves the transient ‘un-LOCK-ing’ of these same domains [7]. So I think that cancer involves the hijacking of a normal mechanism for increasing the variance of gene expression - increased cellular plasticity - that involves these large domains and that is normally involved in processes such as injury response or normal cellular migration. That would go a long way toward explaining things like chemotherapy resistance. Of course the epigenetic changes, although in part probably primary, are also largely driven by mutations during tumor progression [8].

### 4) What have been the most exciting advances in the field in the past couple of years?

There are thousands of papers a year now, so it’s hard to single out particular papers, but there are three exciting areas that are quite new. First are the many discoveries of mutations in chromatin and methylation modifiers in cancer. These appear important in solid tumor progression but may also be initiating events in some leukemias and pediatric solid tumors. Second is the genome-scale analysis of epigenetics in cancer, which has changed our perspective enormously. We now realize that cancer is in large part a disease of the epigenome. In addition to our own work described above, I’m very excited by the results of ENCODE and other large-scale projects that provide potential targets for epigenetic disruptions in cancer. These include the super-enhancers (discovered by Rick Young and others) as well as long RNAs and microRNAs. And of course the new epigenetically driven ependymoma story from the Korshunov-Taylor group [9] is very gratifying, as is the finding from the Yamada group that simply disrupting reprogramming factors during embryogenesis leads to Wilms tumors [10]. It fits our epigenetic progenitor model [11] beautifully.

### 5) Throughout your career you’ve been interested in new ways of thinking about cancer epigenetics. Have you encountered resistance or controversy along the way?

Always. There are two big differences for me between then and now, however. First is my own reaction. I used to get upset with my papers being rejected and now I shrug it off - that’s either maturity or dementia. Second are all the friends I have made in my field who have suffered the same fate. I don’t actually think this is a case of our treating each others’ papers poorly (like the comic book opossum, Pogo, who famously said ‘We have met the enemy and he is us’). Rather, I think there remains a great deal of opposition to translational epigenomics from some members of the classical transcription factor community - it’s not a reach to say so, as they are quite vocal about it.

I think the epigenetic progenitor model [11] changed a lot of people’s minds though. The idea is that cancer involves the polyclonal epigenetic disruption of stem or progenitor cells, and that tumor cells develop on an epigenetically predisposed background. There were certainly early examples of work showing this, such as loss of imprinting in Beckwith-Wiedeman syndrome and common adult tumors, and Jean-Pierre Issa’s work on age-related epigenetic changes in tissues at risk of cancer. The model is strongly supported by the recent discoveries of primary epigenetic cancers, by the reprogramming factor study and by the papers from Teschendorff and colleagues [12] reporting the presence of epigenetically stochastic cells in normal tissue years before tumors develop.

### 6) In 2011 you described increased epigenetic variability in cancer, as well as absolute differences from normal cells. What implications does this have for treating cancer?

So this is what I spend most of my time on these days. Readers should please not confuse my enthusiasm for my ideas with any sense of certainty on my part that they are right. The idea grew from a possible insight into the role of stochasticity in evolution. That is, there would be a selective advantage for genetic variants that increase epigenetic and phenotypic plasticity -aside from mean effects - for genes in which the selective advantage or disadvantage changes unpredictably but recurrently because of a changing environment. In evolution, this would be analogous to something like the availability of nutrients affecting either a positive or negative advantage for size. But in cancer, increased epigenetic plasticity appears to provide the cell with an enormous advantage. The idea is that this allows for rapid selection in a highly changing environment - such as fluctuating oxygen levels or a variable tissue microenvironment [13]. Indeed we have found striking epigenetic variability in at least solid tumors, more so than even the mean changes in epigenetic marks between cancer and normal. This variability appears to have a structural basis as well in the LOCKs and blocks described above [14].

### 7) What challenges need to be addressed for our understanding of the role of DNA methylation in cancer to be translated to clinical application?

At one level - none. I’m not trying to be flippant, but so much of cancer treatment has already been reconsidered mechanistically, and anything that might be helpful to mitigate this terrible disease should at least be considered. Even azacytidine probably acts immunologically rather than on tumor suppressor genes as its main effect. And using epigenetic variability as a risk marker in tissues shows promise in Teschendorff’s work. For therapy, I’m wondering if some of the drugs now in use or development in fact affect the large heterochromatin domains that are destabilized in cancer. If we can develop assays for this, we might be able to develop new lead compounds. For basic science, the most exciting possibility coming down the road is the possibility of a 3D nucleome initiative from the NIH Director’s Common Fund Program, which could relate epigenomics to nuclear structure and function mechanistically at a single-cell level.

### 8) Has understanding the role of DNA methylation in cancer given us insights into the epigenetic basis of other complex diseases?

Absolutely. The approach to integrating genetics, exposure, and gene function that is so central to cancer epigenetics is already bearing fruit in other common disease studies, such as Stephan Beck’s work on diabetes, Tim Spector’s studies of twins, our work on autoimmune disease, and Manel Esteller’s and others’ work on aging.
